# Alteplase for Acute Ischemic Stroke Beyond 3 hours: Enthusiasm Outpaces Evidence

**DOI:** 10.5811/westjem.2021.1.50764

**Published:** 2021-04-02

**Authors:** Ravi Garg

**Affiliations:** Stritch School of Medicine at Loyola University, Department of Neurology, Division of Neurocritical Care, Maywood, Illinois

2019 national guidelines currently make a “strong” recommendation for alteplase treatment for acute ischemic stroke (AIS) in the 3 to 4.5-hour window and a “moderate” recommendation for alteplase treatment guided by magnetic resonance imaging (MRI) in patients with unknown time of symptom onset.^1^ The 3 to 4.5-hour treatment window recommendation is based on a single, randomized clinical trial (RCT): the European Cooperative Acute Stroke Study (ECASS) III.^1^ The recommendation for MRI-based treatment with an unknown time of symptom onset is also based on a single RCT: MRI-Guided Thrombolysis for Stroke with Unknown Time of Onset (WAKE-UP).^1^ Recently, a meta-analysis of RCTs for patients with an unknown time of symptom onset using advanced neuroimaging including the WAKE-UP RCT was published.^2^ This meta-analysis was composed of four incomplete RCTs and concluded alteplase treatment results in better functional outcome at 90 days. Careful consideration of the methodology of these studies should be considered prior to adapting alteplase use beyond the three-hour time window.

## Methodological Limitations of “ECASS III”

In a 2014 editorial, Shy pointed out a statistical error in ECASS III.^3^ The trial’s reported adjusted primary analysis did not account for the baseline imbalance in prior stroke status. The ECASS III authors have not addressed this statistical error in the literature. In a recent publication, Alper et al used the raw data to reanalyze the ECASS III data with appropriate adjustments. In a multivariate model adjusted for both baseline National Institutes of Health Stroke Scale (NIHSS) scores (*P* = .03 between groups) and prior stroke status (*P* = .003 between groups) there was a non-significant difference between alteplase and placebo for all efficacy outcomes.^4^

An unplanned post-hoc reanalysis risks analytical bias by nature. Limitations not considered in the original trial publication, however, support Alper et al’s findings. In the original trial, the authors reported benefit of alteplase treatment for the primary efficacy endpoint: a modified Rankin scale (mRS) score of 0–1. This dichotomization of the mRS includes functional independence with slight disability (mRS = 2) and death (mRS = 6) in the same category. There was no benefit with alteplase treatment, however, for an mRS score of 0–2. There was also no difference in the secondary efficacy endpoint, a “global outcome,” which was the primary endpoint in the NINDS rt-PA RCT.^5^ These results may be explained by the inter-rater reliability of the mRS, which is not uniformly distributed across the scale and is lowest at an mRS of 1.^6^,^7^ Additionally, the fragility index (1) of the primary endpoint is far less than the number of patients lost to follow-up (23).^7^,^8^ The fragility index is relevant given that the method used to handle missing outcome data, worst case imputation, is the method that is most prone to bias in stroke trials.^9^

Finally, ECASS III did not report enrollment of stroke subtypes, which may be an overlooked source of baseline imbalances. For example, in the NINDS rt-PA RCT, 51 patients with small-vessel occlusive disease were randomized into the alteplase arm compared to 30 patients in the placebo arm.^5^ Small-vessel occlusive disease has a significantly better natural history than large-vessel occlusive or cardioembolic stroke subtypes.^5^ Although the authors reported a similar positive effect in favor of alteplase regardless of stroke subtype, the study was not powered to detect these subgroup effects. As shown by Alper et al, small imbalances in a covariate that has a strong relationship with the primary outcome can significantly change the unadjusted effect size. Similar to the NINDS rt-PA RCT, the effect that stroke subtype had on the analysis of ECASS III remains unknown. These limitations of ECASS III make the conclusions from the re-analysis by Alper et al more reliable.

## Methodological Limitations of “Intravenous Alteplase for Stroke with Unknown Time of Onset Guided by Advance Imaging: Systematic Review and Meta-Analysis of Individual Patient Data”

A recent meta-analysis of four incomplete RCTs has been published suggesting that the therapeutic time window for alteplase may be extended further with the use of advanced neuroimaging.^2^ The authors of this meta-analysis concluded that alteplase “resulted in better functional outcome at 90 days than placebo or standard care.” Several considerations should be made prior to accepting the authors’ conclusion. All four individual RCTs included in the meta-analysis were prematurely terminated. Trials that end prematurely risk both type 1 and type 2 errors, and often have efficacy estimates that tend to be biased toward extremes in theory and in practice.^10^–^12^ Meta-analysis composed of underpowered RCTs are unreliable and may be prone to additional bias if clinical heterogeneity is not considered.^13^–^16^ In addition to threats to internal validity, the four incomplete RCTs and meta-analysis have substantially limited external validity due to their inclusion and exclusion criteria.

The four incomplete RCTs enrolled patients with large vessel occlusions (LVO) prior to the publication of multiple RCTs that showed efficacy of mechanical thrombectomy (MT) in patients with LVOs.^17^ Approximately 25% of patients in each arm would be treated differently in current clinical practice by virtue of MT. The authors erroneously conclude that “results of our pooled analysis support treatment with alteplase in patients with large vessel occlusion” without considering that alteplase may not modify the treatment effect of MT. In a meta-analysis of the five pivotal RCTs of MT, there was nearly an identical stratum-specific odds ratio (OR) for both levels of the alteplase stratum compared to the overall treatment effect suggesting alteplase does not modify the effect of MT.^17^ This subgroup-derived hypothesis was confirmed in a recent RCT that found that primary MT is non-inferior to a bridging strategy with alteplase.^18^ Additionally, patients enrolled in late MT-window RCTs were recanalized without bridging alteplase and had comparable outcomes to earlier window trials with a bridging strategy.^19^–^21^

In addition to exclusion of the actual procedure, the more prevalent neuroimaging modality used prior to MT, computed tomography perfusion, was used in only one of four incomplete RCTs included in the meta-analysis. Computed tomography was the favored imaging modality in MT efficacy trials.^22^,^23^ Computed tomography has advantages over MRI in routine clinical practice including increased availability and faster groin puncture times; and MRI may be precluded in patients with cardiac devices or severe agitation.^24^

The meta-analysis also enrolled patients with minor stroke. Trial enrollment in all four incomplete RCTs began prior to the PRISMS RCT, which at the time it was stopped found no signal to benefit with alteplase treatment in 12 efficacy outcomes and strong signal to excess harm.^25^ Considering the natural history of minor stroke, regardless of the designation as disabling or non-disabling, there is hardly a justification for treatment with alteplase without more convincing data.^26^

Finally, one study included in the meta-analysis used a lower dose of alteplase not routinely used in the United States or Europe, and which was not shown to be non-inferior to standard dosing in an RCT enrolling primarily Asian patients.^27^,^28^ Therefore, exclusion of MT, inclusion of minor strokes, and inclusion of a trial that used low-dose alteplase substantially limits the external validity of the results of the pooled analysis.

## Understanding Malpractice Risk and Conclusions

Physicians caring for patients with AIS may be concerned that interpretations of evidence that differ from national guidelines may lead to excess malpractice risk. This is augmented by malpractice data that suggest emergency physicians take a greater malpractice burden compared to neurologists, and withholding alteplase is riskier from a malpractice perspective.^29^ Some misconceptions regarding malpractice risk, however, should be elaborated on. A recent systematic review of acute stroke malpractice found that failure-to-treat cases are frequently merged with failure-to-diagnose cases.^29^ The direct risk of malpractice related to failure to treat alone without failure to diagnose may be further confounded by physicians unilaterally withholding alteplase without informed consent or not documenting conversations regarding informed consent in the medical record.^30^ Although AIS is not considered high risk for litigation occurrence compared to other emergency department diagnoses, mitigation steps such as constructive communication and intelligent documentation are paramount.^31^ Ultimately, stakeholders in acute stroke care should align such that more multi-faceted views can be represented in national guidelines.

Enthusiasm to prevent stroke-related disability may drive more favorable interpretations of the alteplase for AIS literature. Accepting favorable conclusions that are not strongly supported by their respective data should be done so cautiously given the significant risk of intracranial hemorrhage. Methodological pitfalls of the literature should be carefully considered such that enthusiasm does not outpace the evidence.
